# The relationship between CMV reactivation, anti-cytokine treatment and mortality in critical COVID-19 patients

**DOI:** 10.12669/pjms.39.5.7301

**Published:** 2023

**Authors:** Ramazan Gozukucuk, Hasan Huseyin Kilic

**Affiliations:** 1 Ramazan Gozukucuk, Department of Infectious Disease and Clinical Microbiology, Faculty of Dentist, Istanbul Galata University, Evliya Çelebi, Meşrutiyet Cd. No:62, Beyoglu, Istanbul, 34430 Turkey. Hisar Hospital Intercontinental,Saray Mah. Siteyolu Cad.No:7, Umraniye, Istanbul, 34768 Turkey; 2 Hasan Huseyin Kilic, Department of Anesthesiology and Reanimation, Istanbul Dogus Universty Dudullu Yerleşkesi Dudullu Osb Mah. Nato Yolu Cad. 265/ 1 Ümraniye, Istanbul 34775 Turkey. Hisar Hospital Intercontinental,Saray Mah. Siteyolu Cad.No:7, Umraniye, Istanbul, 34768 Turkey

**Keywords:** Tocilizumab, Anakinra, Anti-cytokine treatment, Mortality, Cytomegalovirus (CMV), COVID-19

## Abstract

**Objective:**

To examine the use of anti-cytokine treatment in critical COVID-19 patients and their association with the frequency of CMV cases, viral load level, and mortality in these patients.

**Methods:**

This is a retrospective study. A total of 170 critical and/or intensive care patients with COVID-19 admitted to Hisar Hospital Intercontinental from March 15, 2020, to December 31, 2021 were divided into the use of anti-cytokine treatment group and the no anti-cytokine treatment group. Furthermore, the relationship between CMV reactivation, mortality and anti-cytokine treatment in patients was also examined.

**Results:**

A total of 170 critical COVID-19 patients were included in the study, three of them were excluded. One hundred sixty seven were included in the study of which 38 (22.7%) were found to be CMV DNA positive. As an anti-cytokine treatment, it was observed that tocilizumab was used in 53 patients, anakinra was used in 27 patients, and no anti-cytokine treatment was used in 77 patients. CMV positivity in patients treated with anti-cytokines (31.11%) was found to be significantly higher than in patients who were not treated with it (16.88%) (p:0.033). Furthermore, it was determined that anti-cytokine treatment significantly decreased mortality (p: 0.003) and that there was no significant relationship between CMV reactivation and mortality (p: 0.399).

**Conclusion:**

Even though CMV reactivation was high in critical COVID-19 patients who received anti-cytokine treatment, decrease in mortality were observed with early diagnosis and effective treatment. Therefore, CMV infection should be considered in patients receiving immunosuppressive treatment.

***Clinical Trial Registration***: HisarIH-101/NCT05419206

## INTRODUCTION

Cytomegalovirus (CMV) is a herpes virus and approximately 50-90% of adults are infected. Primary infection is usually asymptomatic in an immunocompetent patient. Since the detection of Coronavirus Disease 2019 (COVID-19), CMV reactivation has also been reported in critically ill patients, but it is unknown whether this reactivation will lead to more adverse outcomes in patients.^1^-^4^ The risk of secondary infection is high in critically ill patients hospitalized with the diagnosis of COVID-19. In particular, long-term hospitalization in the intensive care unit, mechanical ventilation, severe lymphopenia, and immunosuppressive treatments are possible causes of secondary infection.^5^

Among opportunistic infections, fungal infections constitute the highest number of cases in COVID-19 patients. Other reported pathogens are related to viral, bacterial, protozoan, and helminth infections.^6^ Furthermore, in critical COVID-19 patients, cases of COVID-19-associated pulmonary aspergillosis-associated CMV have been reported.^7^ However, there is a scarcity of reported data on CMV infection in COVID-19 patients. Virus-related immune dysfunction occurs in severe COVID-19 patients. For this reason, it has been reported that CMV reactivation may occur, but this situation may not be noticed.^8^,^9^ In critically ill patients, newly developed and untreated infections can lead to rapid aggravation and fatal consequences.

Therefore, it is considered that the diagnosis and treatment of CMV infection may provide positive developments in morbidity and mortality, especially in critical COVID-19 patients receiving immunosuppressive treatment. Hyperinflammation may develop in a part of critical COVID-19 patients. Anakinra, a recombinant interleukin-1 receptor antagonist known to be effective in a variety of hyperinflammatory diseases, and tocilizumab, an interleukin-6 receptor antagonist, are used in this case.^10^,^11^

The relationship between secondary infection and mortality in COVID-19 patients, as well as the use of anti-cytokine treatment and mortality, was examined in different studies.^11^,^12^ In the current study, the use of anti-cytokine treatment, the frequency of CMV cases in these patients, the viral load level, and the relationship between mortality in critical COVID-19 patients were examined.

## METHODS

In this single-center retrospective study, critical and/or intensive care patients followed up in Hisar Hospital Intercontinental with the severe acute respiratory syndrome-COVID-19 coronavirus-2 (SARS-Cov-2) infection between March 15, 2020, and December 31, 2021, were included. During the admission, nasopharyngeal swab samples were taken from all patients for a reverse transcription-polymerase chain reaction (RT-PCR) test (Thermo Fisher / Applied Biosystem Quant Studio-5) to identify COVID-19 SARS-CoV-2 infection. The patient’s records were reviewed retrospectively for the presence of tocilizumab and anakinra in the drugs they used before CMV DNA analysis, and their relationship with CMV reactivation and CMV viral load levels was examined.

Furthermore, the relationship between CMV reactivation and mortality and anti-cytokine treatment in patients was also examined. The severity of the disease was determined according to the World Health Organization criteria, and patients with tachypnea (≥30/min), oxygen saturation in room air <90%, patients with severe clinical condition with bilateral diffuse pulmonary infiltrate, and critical intensive care unit patients who developed acute respiratory distress syndrome (ARDS) were included.^13^ Nucleic acid was isolated from the patient’s material in serum samples, and CMV DNA quantitative viral load levels were studied using the Real-Time PCR method (Anatolia-Bosphore CMV Quantification kit).

### Inclusion criteria

Critically ill patients over the age of 18 diagnosed with COVID-19 and patients followed in the intensive care unit.

### Exclusion criteria

Patients with a mild/moderate course of COVID-19, CMV PCR positive before anti-cytokine treatment, and patients who underwent hematopoietic stem cell transplantation were excluded from the study.

### Statistical Analysis

Descriptive and measurement parameters in COVID-19 case groups with and without anti-cytokines were analyzed in the SPSS Statistics Version 25 Program. CMV positive percentages and mortality of the groups were evaluated with Chi-Square Test, and differences in CMV DNA viral load were evaluated with one-way ANOVA. The statistical p-value of <0.05 was considered significant.

### Ethical Approval

The Ethics Committee of the Hisar Intercontinental Hospital approved this study (04.04.2022/22-22).

## RESULTS

The study included 170 critical and/or intensive care patients with the diagnosis of COVID-19 were admitted in the hospital. Three were excluded because they were with a history of hematopoietic stem cell transplantation. Of them, 167 were included in the study. The mean age of the patients was 59.2 (26-91 years), 63.7 years for women, and 58.6 years for men and 24 of whom were female (14%), and 143 of whom were men. Ninety (53.9%) patients (Group-2) were using anti-cytokines drugs, and 77 (46.1%) patients (Group-1) did not have any anti-cytokines drugs. Considering the use of anti-cytokines in these patients, it was found that 53 received tocilizumab (Group-3), 27 received anakinra (Group-4), and 10 received both (Group-5). CMV DNA was positive (Group-6) in 38 (22.7%) and negative (Group-7) in 129 (77.3%) of the patients included in the study. It was determined that the two patient groups were similar in age, comorbidity status, and corticosteroid use (p:0.266). CMV positivity was found to be significantly higher in Group-2 (31.11%) compared to Group-1 (16.88%) who did not receive anti-cytokine treatment (p:0.033) (Table-I & II).

**Table-I:** CMV positivity rate, CMV DNA viral load and mortality values in COVID-19 cases with and without anti-cytokine therapy

COVID-19 Cases (N=167)	Age (Years) mean±SD	Gender (M/ F)	CMV DNA (IU/mL) mean±SD	Mortality %	CMV Positive %
Group-1 (No Anti-cytokin) n =77	61.3±16.1	62/15	19044 ± 35256 ^a^	37 (48.05 %) ^b^	13 ( 16.88 %) ^d^
Group-2 (TCZ and/or, AKR) n=90	57.1 ±13.1	81/9	13891± 28437	23 (25.50 %)	28 ( 31.11 %)
Group-3 (TCZ) n=53	56.9 ±12.6	46/7	12231 ± 22219	12 (22.64 %)	17 ( 32.07 %)
Group-4 (AKR) n=27	57.4 ±13.5	26/1	5517 ± 7092	8 (29.63 %)	11 (39.29 %)
Group-5 (TCZ with and AKR) n=10	56.8 ±9.9	10/0	19303 ± 6650	3 (30.00 %)	4 ( 44.44 %)
Group-6 (CMV Positive) n=38	57.4±13.6	31/7	13669 ± 24763	10 (24.39 %) ^c^	-
Group-7 (CMV Negative) n=129	59.5±15.0	112/17	-	36 (27.48 %)	-

*TCZ:*Tocilizumab, *AKR:* Anakinra

a p:0.513, (p>0.05; There is no significant difference between CMV viral loads of COVID-19 groups with and without anticytokines

b p:0.003, (p<0.01; Mortality is very significantly higher in COVID-19 cases without anticytokines

c p:0.399, (p>0.05; There was no significant difference in mortality in CMV positive COVID-19 cases

d p:0.033, (p<0.05; There is significant difference between CMV positivity rates of COVID-19 groups with and without anticytokines

**Table-II:** Chi-Square significance values of CMV DNA positivity rates in COVID-19 cases with and without anti-cytokine therapy.

	CMV Positive %		Mortality %	
	Chi-Square value	p	Chi-Square value	p
Group 1 vs-Group 2 (No Anticytokin-TCZ and/or, AKR)	x^2^=4.535	p: 0.033 ^a^	x^2^=9.123	p: 0.003 ^b^
Group 1 vs-Group 3 (No Anticytokin - TCZ)	x^2^=4.082	p: 0.043 ^a^	x^2^=8.631	p: 0.003 ^b^
Group 1 vs-Group 4 (No Anticytokin - AKR)	x^2^=6.410	p: 0.011 ^a^	x^2^=2.761	p: 0.096
Group 1 vs -Group 5 (No Anticytokin-TCZ with and AKR)	x^2^=3.008	p: 0.083	x^2^=1.161	p: 0.281
Group 3 vs -Group 4 (TCZ - AKR)	x^2^=0.590	p: 0.442	x^2^=0.466	p: 0.495

*TCZ:*Tocilizumab, *AKR:* Anakinra ;

a (p<0.05),

b (p<0.01); The statistical p-value of <0.05 was considered significant.

**Fig.1:**
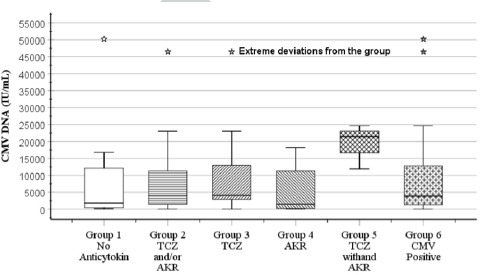
CMV viral load box plot of COVID-19 cases with and without anti-cytokine therapy.

CMV viral load levels in the patients were found to be between 32 and 35823 IU/mL, and no significant difference was found in viral load levels between the two groups with and without anti-cytokine treatment (p:0.513) (Fig.1 & Table-I). Furthermore, the mortality was 27.48% in CMV-negative critical COVID-19 patients, while the mortality rate was lower in CMV-positive patients (24.39%), contrary to expectations, but there was no statistically significant difference (p:0.69) (Table-I). As an important finding, the use of anti-cytokine treatment was found to significantly reduce mortality in COVID-19 (p: 0.003) (Table-I & II).

## DISCUSSION

The analysis of this study showed that the rate of CMV positivity was significantly higher in critical COVID-19 patients who received anti-cytokine treatment than in patients who did not. There is a scarcity of reported data on CMV infection in COVID-19 patients. It has been reported that virus-related immune dysfunction occurs in critical COVID-19 patients, and then CMV reactivation may occur, but this situation may not be noticed.^8^,^9^ It has been reported that the use of corticosteroids in COVID-19 patients is associated with the risk of CMV reactivation.^14^-^17^ In another study where it was reported that the use of long-term steroid treatment may increase the risk of coinfection such as candidemia or CMV pneumonia, the mean age of the patients was 60, and 48% of the patients were female.^18^ The mean age of the patients who received anti-cytokine treatment and did not in our study was 59.2 years, and there was no difference in terms of age, comorbidity status, or corticosteroid use, but unlike other studies, only 14% of the patients were female.

The risk of secondary infection is high in critically ill patients hospitalized with the diagnosis of COVID-19. Lymphopenia develops, especially in severe SARS-CoV-2 infections, resulting in cellular immune system deficiencies and eventually increasing the risk of re-infection or reactivation with CMV.^12^ It has been reported that CMV infection may predispose patients to severe cases of COVID-19 by disrupting T cell differentiation and upregulating inflammatory cytokines.^9^

As is known, T cells are essential very important in the regulation of immune responses against viral infections. Similar to COVID-19 patients, people infected with CMV usually have higher serum levels of IL-6.^12^ In contrast, the use of tocilizumab as a monoclonal antibody against IL-6 is effective in the treatment of COVID-19 patients in the intensive care unit. Corticosteroids and monoclonal antibodies have been used as an immunosuppressive treatment to alleviate cytokine storms in critical COVID-19 patients, and these treatments have been reported to reduce mortality. Similar to other studies, mortality was found to be significantly lower in our patients receiving anti-cytokine treatment. However, CMV cases have been reported among COVID-19 patients receiving corticosteroid or tocilizumab treatments.^9^,^12^,^19^,^20^

In our study, CMV positivity was found to be significantly higher in the patients receiving anti-cytokine treatment compared to the patients who did not receive it (p<0.05). In critical COVID-19 patients, it is thought that coinfection with CMV may worsen the underlying disease and induce cytokine storms.^19^ Accordingly, early detection of coinfections is important because of their serious adverse effects on treatment and prognosis. Early diagnosis of opportunistic infections in COVID-19 patients will lead to early treatment and ultimately lower morbidity and mortality.^21^

The early diagnosis of CMV and its effective treatment resulted in an improvement in mortality in the critically ill patients we followed up. It has been shown in different studies that CMV viral load and mortality are related after hematopoietic stem cell transplantation.^22^-^25^ Although anti-cytokine treatment increased CMV reactivation in our patients, viral load levels did not increase significantly. Furthermore, there was no significant difference between CMV-positive and non-positive patients in terms of mortality.

We report that a high proportion of patients diagnosed with COVID-19 and receiving anti-cytokine therapy have CMV reactivation. It is a rarely reported condition in the literature. The clinical significance of these results is that early detection of opportunistic infections in patients will lead to early treatment and ultimately lower morbidity and mortality.

### Limitations

Because it is a retrospective study, there are limitations as the desired tests cannot be performed in every patient and CMV PCR is not a standard protocol test that should generally be performed on all COVID-19 patients. Therefore, a selection bias may have occurred, which may have an impact on the results.

## CONCLUSIONS

In the current study, the use of anti-cytokine treatment, the frequency of CMV cases in these patients, the viral load level, and the relationship between mortality in critical COVID-19 patients were examined. As a result, the CMV reactivation risk was significantly higher in patients receiving anti-cytokine treatment, whereas mortality was significantly lower in patients receiving effective treatment. Therefore, it is thought that routine monitoring for CMV infection may provide positive developments in morbidity and mortality with early diagnosis, especially in critical COVID-19 patients receiving intense immunosuppressive treatment.

### Authors’ Contributions:

**RG:** Conceived and designed the study.

**RG** and **HHK:** Collected the data and performed the analysis.

**RG:** Was involved in the writing of the manuscript and is responsible for the integrity of the study.

All authors have read and approved the final manuscript.
